# Comparison of Virtual Dose Simulator and K-Factor Methods for Effective Dose Assessment in Thoracic CT

**DOI:** 10.3390/tomography11110128

**Published:** 2025-11-13

**Authors:** Roch Listz Maurice

**Affiliations:** 1 Groupe Biomédical Montérégie, Centre Intégré de Santé et des Services Sociaux de la Montérégie-Centre, Brossard, QC J4W 3J8, Canada; roch-listz.maurice.cisssmc16@ssss.gouv.qc.ca; 2 Centre de Recherche Charles-Le Moyne, Centre Intégré de Santé et des Services Sociaux de la Montérégie-Centre, Longueuil, QC J4V 2G9, Canada

**Keywords:** effective dose (ED), K-factor, virtual dose simulator, computed tomography (CT), medical imaging, radiation dose, patient dose optimization, patient radiation safety, individual cumulative dose monitoring, population dose assessment

## Abstract

**Rationale and Objective:** Medical imaging, particularly computed tomography (CT), is the largest man-made contributor to collective radiation exposure. This study compares methods for assessing CT radiation dose, focusing on thoracic examinations. **Population investigated:** We retrospectively analyzed 3956 non-contrast thoracic CT exams from 1553 females (mean age 70 ± 12 years) and 2403 males (mean age 69 ± 12 years). **Methods:** Data were acquired using a Siemens Somatom Force CT-Scanner (installed in 2015). Exposure parameters and patient somatic data were recorded and used as inputs for the Virtual Dose Simulator (VDS), which served as the gold standard for effective dose (ED^ref^) measurement. Additionally, ED was calculated using two ICRP-103 K-factor methods: Shrimpton et al. (ED^shr^) and Romanyukha et al. (ED^rom^). **Results:** Regression analysis demonstrated strong linear relationships between ED^ref^ and both weight and BMI (R^2^ ≥ 0.84), with ED^ref^ values ranging from 1.55 to 4.59 mSv. Even stronger linear relationships were observed between ED^ref^ and CT scanner tube current, particularly for women (R^2^ = 0.93) and men (R^2^ = 0.90). Similar trends emerged for dose-length product (DLP), which showed high correlations for both women (R^2^ = 0.95) and men (R^2^ = 0.94). Compared to VDS, ED^rom^ underestimated women’s doses by 10% and slightly overestimated men’s doses by 1%, while ED^shr^ underestimated the effective dose by 18% for women and 9% for men. **Conclusion:** This study demonstrates that K-factor methods provide a simple, efficient, and clinically practical approach for both individual cumulative dose monitoring (critical for patients requiring repeated imaging) and population-level dose assessment (essential for epidemiological risk evaluation). The high reliability of K-factor-based estimates, as demonstrated in this work, underscores their potential for integration into clinical practice to enhance dose optimization and patient safety.

## Simple Summary

Computed tomography (CT) is the largest human-made source of population-wide radiation exposure, yet accurately estimating patient dose remains a major challenge. While Monte Carlo simulations are considered the gold standard, they are too complex for routine clinical use. This study retrospectively analyzes 3956 non-contrast thoracic CT scans from 1553 female and 2403 male patients, comparing Monte Carlo simulations with simpler K-factor methods. The results clearly demonstrate that K-factor methods provide a reliable, practical, and accessible alternative. K-factors enable both individual patient dose tracking—essential for those requiring repeated imaging—and broader population-level risk assessment, supporting their immediate integration into clinical practice for improved radiation safety.

## 1. Introduction

The World Health Organization (WHO) estimates that medical use of radiation accounts for approximately 98% of the population’s exposure from all artificial sources and represents around 20% of total radiation exposure (including natural background radiation) [1]. Globally, more than 4.2 billion diagnostic radiology examinations, 40 million nuclear medicine procedures, and 8.5 million radiotherapy treatments are performed annually [1,2].

Although ionizing radiation provides significant health benefits such as in medical imaging and cancer radiotherapy, even controlled doses carry potential risks that include an increased likelihood of cancer development after a latency period of years to decades [3]. Computed tomography (CT), in particular, is a major contributor to medical radiation exposure, prompting numerous studies to evaluate the lifetime attributable risk of radiation-induced cancer from CT scans [4,5,6,7].

The effective dose (ED) is a key metric in medical imaging, providing a standardized estimate of stochastic risks (e.g., cancer, hereditary effects) from ionizing radiation exposure [8]. For high-precision applications, Monte Carlo simulation-based methods are considered the gold standard for organ dose estimation, requiring detailed knowledge of scanner parameters, examination protocols, and patient anatomy [9]. In contrast, simplified methods, such as the K-factor, offer a practical alternative for estimating ED with minimal procedural details and without scanner-specific modeling [10].

The quest for methods that balance accuracy and efficiency for estimating effective dose in CT is a persistent challenge in medical physics [11]. While Monte Carlo simulations are considered the gold standard, they remain unsuitable for routine clinical use due to their computational intensity [12]. In response, methods using conversion factors (K-factors) between the Dose-Length Product (DLP) and effective dose have become established for population-based dose monitoring, as endorsed by organizations like the AAPM [13].

The widely used approach based on the model by Shrimpton et al. [14] represents a foundational methodology grounded in historical computational anthropomorphs. Alongside this, alternative conversion factors have been developed using more advanced anatomical phantoms, such as those proposed by Romanyukha et al. [15]. Comparative studies have begun to evaluate these different methodologies [16], yet a focused evaluation in the context of non-contrast thoracic CT, benchmarking them against an advanced Monte Carlo reference (VirtualDose) to quantify their relative performance, is still lacking. This study therefore aims to fill this gap by comparing Monte Carlo simulations vs. K-factors, focusing on non-contrast thoracic CT examinations.

## 2. Methods

### 2.1. ED: From Derivation to Implementation

#### 2.1.1. Theoretical Framework for CT Dosimetry

This section summarizes the fundamental concepts of radiation dosimetry necessary to contextualize the K-factor and VirtualDose methods compared in this study. The absorbed dose (D_T_, in Gy) quantifies the energy deposited per unit mass and is influenced by scanner parameters (e.g., kVp, mA) and patient size [8,17,18,19,20]. In CT, standardized metrics are used to report radiation output, but require conversion for patient-specific estimates. The three key parameters are:

(a) CTDIvol (Volume-averaged Computed Tomography Dose Index): Represents the average absorbed dose in a standardized acrylic phantom (16 cm for head or 32 cm for body scans) during a single axial rotation. It accounts for scanner output and pitch but does not consider patient-specific anatomy [21].
(b) CTDIw (Weighted CTDI): Combines central and peripheral phantom measurements to approximate the dose distribution across the phantom’s cross-section [21].
(c) DLP (Dose-Length Product, in mGy·cm): Calculated as CTDIvol × scan length, this reflects the total radiation output of the scan. However, conversion factors (e.g., K-factors) are required to estimate organ-specific D_T_ or effective dose (ED) [10].

The biological impact is assessed through the equivalent dose (H_T_, in Sv), calculated as:

(1) $$H_{T}=w_{R}\cdot D_{T}\;\;\;\;$$

where w_R_ is the radiation weighting factor (w_R_ = 1 for X-rays) [8]. The effective dose (ED, in mSv) integrates organ doses weighted by tissue radiosensitivity (w_T_) to estimate stochastic risk:

(2) $$\begin{array}{ll} E_{D} & =\sum_{T}w_{T}\cdot H_{T}\\ & =\sum_{T}w_{T}\cdot w_{R}\cdot D_{T}=\;w_{R}\sum_{T}w_{T}\cdot D_{T}\; \end{array}\;\;\;\;$$

This framework underpins the dose comparisons presented in this work.

#### 2.1.2. Monte Carlo Simulation-Based Methods in CT Dosimetry

Monte Carlo (MC) methods are considered the gold standard for high-precision organ dose estimation, as they simulate radiation transport through detailed anatomical models [9]. While their full theory is beyond this paper’s scope [22,23,24], they provide the foundational principle for the VirtualDose simulator used in this study.

#### 2.1.3. Virtual Dose CT Simulator

The VirtualDose™ CT (VDCT) simulator(Virtual Phantoms Inc., Albany, NY 12205-5681, USA) is a computational tool designed to estimate radiation dose exposure in patients undergoing CT scans. It combines Monte Carlo simulations with an extensive library of anatomical patient phantoms [25]. The platform’s library, detailed in Gao et al. [26], encompasses a wide range of models, including:

Adults of varying body weights and heights

Overweight and obese adults

Children of different ages and body sizes

Pregnant females at three gestational stages.

For this study, the simulator automatically selected the most appropriate phantom for each dose calculation based on the specific somatic data (weight, height) and scan parameters, following its internal patient-matching protocol to generate the reference effective dose (ED^ref^) values.

#### 2.1.4. K-Factor-Based Method in CT Dosimetry

Unlike complex MC methods, the K-factor provides a practical alternative for estimating effective dose (ED) with minimal procedural details and without requiring scanner-specific modeling [10]. In CT imaging, this method employs a conversion factor to estimate organ dose or effective dose from the dose-length product (DLP) or CT dose index (CTDI). The effective dose (ED) is calculated as follows [13]:

(3) $$E_{D}=K\cdot DLP$$

where

ED = Effective dose (mSv)

K = K-factor (mSv·mGy^−1^·cm^−1^)

DLP = Dose-length product (mGy·cm)

The K-factor varies depending on the anatomical region due to differences in tissue composition and radiation sensitivity [8,13,18,27]. For thoracic CT examinations, as in this study, we compared two ICRP-103 K-factor methods: Shrimpton et al. (K = 0.019) [14] and Roman et al. (K = 0.021) [15].

It is important to note that the ED estimate derived from Equation (3) carries a combined uncertainty that propagates through the simplified conversion chain (CTDIvol → DLP → K-factor → ED). This uncertainty stems from the inherent limitations of the CTDIvol metric (e.g., its dependence on phantom size and scan geometry), the DLP calculation, and the application of a generic K-factor. The K-factor values themselves are typically derived from simulations using standardized reference phantoms and do not account for patient-specific attributes such as individual size, gender, or exact anatomical makeup. While this approach offers valuable practicality for dose comparison and tracking, these uncertainties should guide the interpretation of the results, underscoring that ED values from this method are population-based estimates for reference purposes. In contrast, the reference method used in this study (VirtualDose) incorporates patient-somatometric data to provide a more tailored estimation, thereby mitigating some of these uncertainties related to anatomical representation.

### 2.2. Study Population and Clinical Context

#### 2.2.1. Clinical Utility of Chest CT

Chest CT is a cornerstone of thoracic diagnosis. Among many applications, it is primarily used to detect lung cancer and metastasis to the thorax [28,29], as a first-line imaging for suspected pulmonary embolism [30], and to assess infectious diseases such as pneumonia [31], tuberculosis, and fungal infections [32].

#### 2.2.2. Global Utilization Patterns of Chest CT

Chest CT ranks among the top 3 most frequent CT examinations worldwide, though regional disparities exist [33,34,35]. In the U.S., it accounts for ~25% of all CT scans, driven by lung cancer screening (low-dose protocols) [36] and CT Pulmonary Angiography (CTPA) for pulmonary embolism [37]. Similar trends are observed in Europe, with heavy utilization in lung cancer screening programs [38] and oncology staging (e.g., metastatic workup) [39].

#### 2.2.3. Chest CT Dosimetry

When considering single-phase diagnostic CT exams (excluding angiography and multiphase protocols), standard chest CT, with a typical effective dose estimate of 5–7 mSv [34,40], ranks among the top 5 most irradiant routines. This remains significantly below common reference levels for other regions: abdomen/pelvis (8–10 mSv) [18,33,41], spine (6–10 mSv) [40], pelvis-alone (6–8 mSv) [41], and whole-body CT (10–15 mSv) [34,40].

#### 2.2.4. Chest CT Population Dose Burden

Combining examination frequency (top 3 most performed) and dosimetry (top 5 most irradiant), chest CT ranks #2 in global population dose burden [33,40], due to its high utilization (~20–25% of all CTs), and moderate per-exam dose (5–7 mSv), surpassing spine CT in collective impact despite lower per-scan doses.

#### 2.2.5. Materials

CT imaging was performed using a Siemens Somatom Force CT scanner (Siemens Healthineers, Erlangen, Germany, installed in 2015). This system was classified as an “advanced imaging” device, the highest technological category defined by ECRI [42].

Patient dose and exposure parameter data were obtained from Radiation Dose Structured Reports (RDSR), automatically generated and stored in the PACS (Picture Archiving and Communication System) of the Radiology Department. The RDSR, a DICOM-compliant text file, contains comprehensive metadata related to CT examinations, including exposure parameters. Somatic data (e.g., patient demographics, clinical indications) were retrieved from the Radiology Information System (RIS).

The extracted exposure and somatic data were merged into a structured Microsoft Excel^®^ (Microsoft 365 MSO) database for statistical analysis. Effective dose (ED) calculations were performed using two methods:

ED^ref^ was computed using the VDCT simulator (VirtualDoseCT);

ED^shr^ and ED^rom^ were derived via Excel-based calculations using the K-factors published by Shrimpton et al. [14] and Romanyukha et al. [15], respectively.

Exclusion criteria encompassed scans performed with:

Dual-energy source CT;

Non-spiral (sequential) acquisition protocols;

Contrast-enhanced thoracic studies.

## 3. Results

### 3.1. Ethical Framework and Data Source

This work was funded by the Quebec Ministry of Health and Social Services (“Ministère de la Santé et des Services Sociaux du Québec (MSSSQ)”) through its annual operating budget as part of its ongoing quality improvement initiatives. The legal basis for this study is provided by Articles 1 and 2 of Chapter I of the Public Health Act (“S-2.2—Loi sur la santé publique”), which authorizes the MSSSQ to use patient data for public health surveillance when the population’s health is potentially threatened [43], as is the case with medical imaging radiation. Consequently, for this retrospective analysis, neither individual patient consent nor specific ethics committee approval was required.

All data were sourced from a single, high-volume tertiary care institution within the MSSSQ network. To ensure strict confidentiality, all patient data—specifically the Radiation Dose Structured Reports (RDSR)—were thoroughly anonymized prior to analysis, with all identifying information for patients and healthcare personnel permanently removed.

### 3.2. Study Cohort and Imaging Data

This retrospective study analyzed a consecutive series of 3956 non-contrast thoracic CT exams, including 1553 female patients (mean age 70 ± 12 years) and 2403 male patients (mean age 69 ± 12 years). All scans were performed on a single Siemens Somatom Force CT scanner (Siemens Healthineers, Forchheim, Germany) located at the aforementioned institution, over a six-year period from January 2017 to December 2022.

For the purpose of this analysis, the population was partitioned by sex and into distinct Body Mass Index (BMI) subgroups. These subgroups were defined as a sequence of half-open intervals, covering the range from 15 to 47 kg/m^2^. Formally, the set of all BMI intervals I is defined by the union:

(4) $$I=\underset{n}{⋃}\;[\begin{array}{cc} n & n+2 \end{array}[,\;n\in \;\left\{15,\;17,\;19,...\right\}\;\;$$

As illustrated in Table 1, the female and male populations were distributed across these predefined subgroups. The distribution of patients across these BMI subgroups was non-uniform, a finding consistent with the known epidemiological distribution of BMI in human populations [44,45], further shaped by the health-status bias inherent in a clinical cohort undergoing thoracic CT imaging [46]. Specifically, the presence of patients in both low-BMI (e.g., potentially reflecting cachexia or chronic illness) and high-BMI (e.g., reflecting obesity-related comorbidities) subgroups aligns with the J-shaped relationship often observed between BMI and morbidity [47].

For validation purposes, the effective dose reference (ED^ref^) data are first analyzed in relation to patient somatic and exposure parameters, respectively. Subsequently, a comparison is made between the ED^ref^ and K-factor-based methods, specifically ED^shr^ (Shrimpton et al. [14]) and ED^rom^ (Romanyukha et al. [15]).

### 3.3. Effective Dose Reference (ED^ref^) Data

#### 3.3.1. ED^ref^ and Patient Somatic Data

**Figure 1:**
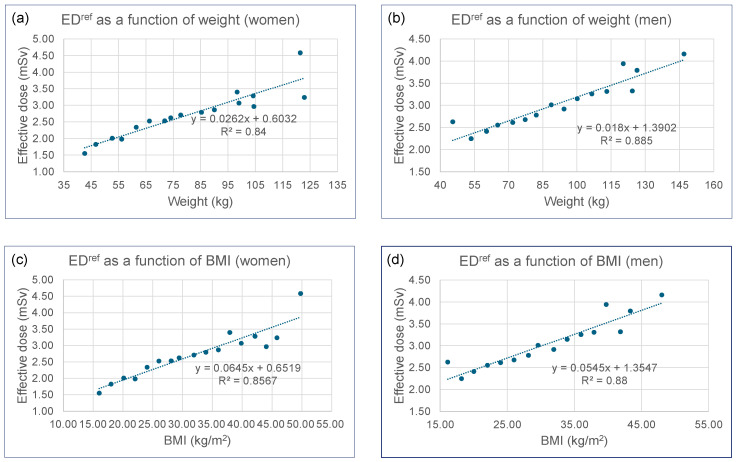
Plots of ED^ref^ as a function of: (**a**) body weight for women; (**b**) body weight for men; (**c**) BMI for women; (**d**) BMI for men.

Regression analyses demonstrated very strong and highly significant linear relationships between ED^ref^ and body parameters. For all reported regression models, the 95% confidence intervals for the coefficients are provided alongside the point estimates to quantify the precision of the relationships. For women, ED^ref^ (mSv) = 0.0262 [0.020, 0.032] × weight (kg) + 0.6032 [0.072, 1.134] (R^2^ = 0.84 and *p* < 10^−6^, Figure 1a) and ED^ref^ = 0.0645 [0.050, 0.079] × BMI (kg/m^2^) + 0.6519 [0.164, 1.140] (R^2^ = 0.86 and *p* < 10^−6^, Figure 1c). For men, ED^ref^ = 0.0180 [0.014, 0.022] × weight (kg) + 1.3902 [1.032, 1.749] (R^2^ = 0.89 and *p* < 10^−7^, Figure 1b) and ED^ref^ = 0.0545 [0.043, 0.066] × BMI (kg/m^2^) + 1.3547 [0.981, 1.729] (R^2^ = 0.88 and *p* < 10^−7^, Figure 1d). The high R^2^ values (0.84–0.89) indicate that body weight and BMI account for 84–89% of the variance in ED^ref^ values. Women exhibited steeper regression slopes (0.0262 vs. 0.0180 mSv/kg), indicating greater ED^ref^ increase per unit weight gain, while men had higher intercept values (1.3902 vs. 0.6032 mSv), which may reflect sex-specific anatomical differences. Overall, ED^ref^ values ranged from 1.55 to 4.59 mSv across the cohort.

#### 3.3.2. Distribution and Regression Robustness

**Figure 2:**
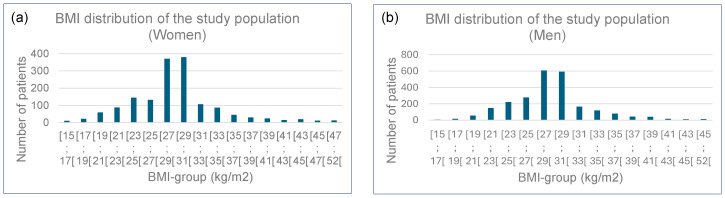
Histograms of BMI: (**a**) for women; (**b**) for men.

The body mass index (BMI) distribution of the study population is presented in Figure 2, visually illustrating the non-uniform distribution of patients across the predefined BMI intervals. The potential influence of this sample distribution on the regression analysis was considered in the methodological design. To mitigate the impact of sample imbalance and outliers, the regression analysis was deliberately performed on the median values calculated for each BMI subgroup. This approach inherently reduces the bias that could arise from under-represented extreme bins, as the median, by definition, is robust to extreme values and uneven sample sizes. It ensures that the calculated slopes reflect central trends across the BMI spectrum rather than being disproportionately influenced by individual outliers or sparsely populated bins. Therefore, we are confident that the strong linear relationships observed (R^2^ > 0.84) robustly characterize the primary dependence of ED_ref_ on body size parameters within our cohort.

#### 3.3.3. ED^ref^ and Exposure Parameters

**Figure 3:**
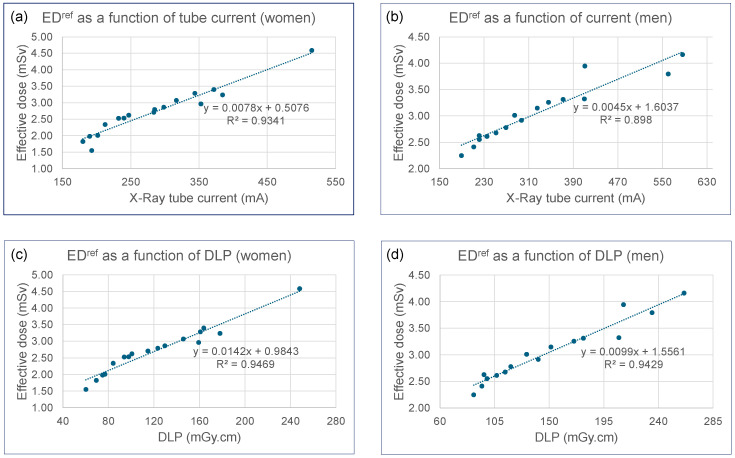
Plots of ED^ref^ as a function of: (**a**) CT scanner tube current for women; (**b**) CT scanner tube current for men; (**c**) DLP for women; (**d**) DLP for men.

Regression analyses demonstrated very strong and highly significant linear relationships between ED^ref^ and CT acquisition parameters. For all reported regression models, the 95% confidence intervals for the coefficients are provided alongside the point estimates to quantify the precision of the relationships. For women, ED^ref^ (mSv) = 0.0078 [0.007, 0.009] × tube current (mA) + 0.5076 [0.169, 0.846] (R^2^ = 0.93 and *p* < 10^−9^, Figure 3a) and ED^ref^ = 0.0142 [0.012, 0.016] × DLP (mGy.cm) + 0.9843 [0.741, 1.227] (R^2^ = 0.95 and *p* < 10^−10^, Figure 3c). For men, ED^ref^ = 0.0045 [0.004, 0.005] × tube current (mA) + 1.6037 [1.308, 1.899] (R^2^ = 0.90 and *p* < 10^−7^, Figure 3b) and ED^ref^ = 0.0099 [0.009, 0.011] × DLP (mGy.cm) + 1.5561 [1.333, 1.779] (R^2^ = 0.94 and *p* < 10^−9^, Figure 3d). Women consistently exhibited steeper slopes across all parameters, while men showed higher baseline values. These preliminary data (Figure 1 and Figure 3), which demonstrate strong correlations between ED^ref^ and both patient somatic parameters and scanner exposure settings, establish a robust foundation for comparing the VDCT simulator with K-factor-based methods.

### 3.4. Comparison with K-Factor-Based Methods

**Figure 4:**
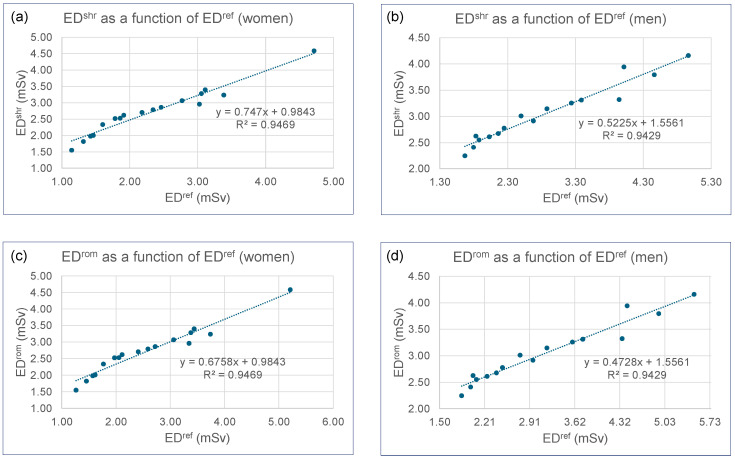
Plots of: (**a**) ED^shr^ as a function of ED^ref^ for women; (**b**) ED^shr^ as a function of ED^ref^ for men; (**c**) ED^rom^ as a function of ED^ref^ for women; (**d**) ED^rom^ as a function of ED^ref^ for men.

Figure 4 compares ED^ref^ with ED^shr^ and ED^rom^. Regression analyses revealed very strong and highly significant linear relationships for both women and men (R^2^ ≥ 0.94). The regression equations demonstrate systematic underestimation patterns: for women, ED^shr^ = 0.747 × ED^ref^ + 0.9843 (R^2^ = 0.95 and *p* < 10^−10^, Figure 4a) and ED^rom^ = 0.676 × ED^ref^ + 0.9843 (R^2^ = 0.95 and *p* < 10^−10^, Figure 4c); for men, ED^shr^ = 0.523 × ED^ref^ + 1.5561 (R^2^ = 0.94 and *p* < 10^−9^, Figure 4b) and ED^rom^ = 0.473 × ED^ref^ + 1.5561 (R^2^ = 0.94 and *p* < 10^−9^, Figure 4d). The consistent intercepts across methods reflect the constant K-factors used in ED^shr^ and ED^rom^ calculations. When compared to the reference method (ED^ref^), ED^shr^ underestimated the effective dose by 18% for women and 9% for men, while ED^rom^ underestimated women’s doses by 10% and slightly overestimated men’s doses by 1%.

To further evaluate the agreement between the Monte Carlo-based reference method (ED^ref^) and the K-factor-derived estimates (ED^rom^, ED^shr^), Bland–Altman analyses were performed [48]. This method assesses agreement between two quantitative measurements by plotting the difference between the methods against their mean, and calculating the mean bias and the 95% limits of agreement, with:

Mean bias (MB) represents the average difference (ED^ref^ − ED^shr^ or ED^ref^ − ED^rom^, respectively) and SD is the standard deviation of these differences.

The Lower Limit of Agreement (LLA) and Upper Limit of Agreement (ULA) were calculated according to the standard Bland–Altman method as follows:

LLA = MB − 1.96 × SD and ULA = MB + 1.96 × SD(5)

**Figure 5:**
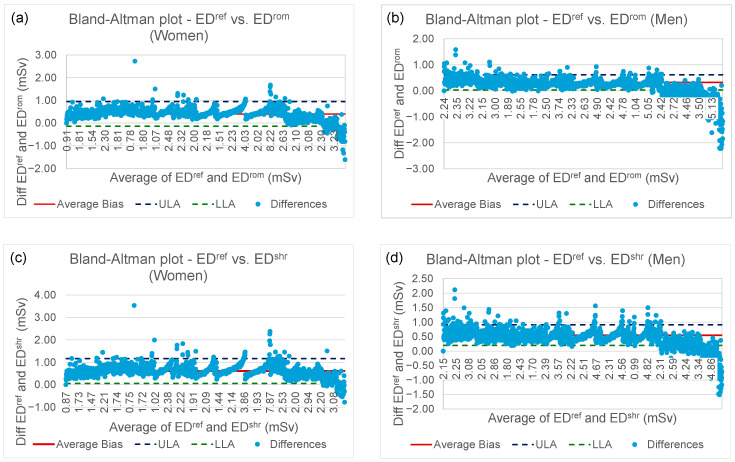
Bland–Altman plots of: (**a**) ED^ref^ vs. ED^rom^ (Women); (**b**) ED^ref^ vs. ED^rom^ (Men); (**c**) ED^ref^ vs. ED^shr^ (Women); (**d**) ED^ref^ vs. ED^shr^ (Men). LLA: Lower Limit of Agreement; ULA: Upper Limit of Agreement; Differences: Point-by-point difference between ED^ref^ and, respectively, ED^rom^ and ED^shr^.

The plots (Figure 5) visualize these metrics for the entire cohort.

The analysis for ED^ref^ versus ED^rom^ revealed mean biases of +0.4042 mSv and +0.3242 mSv, for women and men, respectively, indicating that ED^ref^ systematically provided slightly higher values. The 95% limits of agreement ranged from −0.1434 to +0.9518 mSv and +0.0333 to +0.6150 mSv, for women and men, respectively, demonstrating the potential range of discrepancies for individual dose estimates.

Similar trends were observed for ED^ref^ versus ED^shr^ with mean biases of +0.6097 mSv and +0.5534 mSv, for women and men, respectively, while limits of agreement ranged from +0.0517 to +1.1676 mSv and +0.1972 to +0.9096 mSv, respectively. The widths of these limits underscore that while the K-factor methods are valuable for population-level estimates, their use for patient-specific dose estimation should consider this inherent variability.

## 4. Discussion

### 4.1. Potential Benefits of K-Factor Methods

When compared to the reference dose values derived from VirtualDoseCT—considered the gold standard in this study—the results demonstrate that K-factor methods provide a computationally efficient alternative for estimating effective dose in thoracic CT, but with varying levels of accuracy. The method proposed by Romanyukha et al. (ED^rom^) showed acceptable reliability for clinical use in our cohort, particularly for men, where it induced a minimal median overestimation of +1%. In contrast, the Shrimpton-based factor (ED^shr^) led to a more systematic underestimation, especially pronounced in women (−18%). This sex-dependent discrepancy underscores that the “benefit” of a K-factor is intrinsically tied to its specific value and its applicability to patient subpopulations.

The underestimation observed with the ED^shr^ method, and to a lesser extent with ED^rom^ in women, can be explained by thoracic anatomy. The breast region, a tissue of high radiosensitivity (with a tissue weighting factor of 0.12 according to ICRP 103 [8]), contributes significantly to the effective dose. The more pronounced underestimation with ED^shr^ suggests that this model, being based on a historical computational framework using mathematical phantoms and a narrower range of scanner models, does not fully capture these effects in its conversion coefficients, unlike modern simulations employing voxelized phantoms and updated beam models.

The superior performance of the ED^rom^ method in our cohort indicates that its conversion factors provide a more accurate representation of the dose delivered to female anatomy. This improvement can plausibly be attributed to more advanced modeling that better integrates the contribution of breast tissue, both in terms of its radiosensitivity and its attenuation effects.

The strong linear correlations observed between the reference dose (EDref) and both tube current (R^2^ ≥ 0.90) and DLP (R^2^ ≥ 0.94) reinforce the fundamental principle behind K-factors: DLP is a robust surrogate for effective dose. This strong relationship validates the use of DLP-based methods for protocol optimization and compliance with Diagnostic Reference Levels (DRLs) in a clinical audit context. The K-factor approach, as exemplified here by ED^rom^, offers the computational simplicity needed to facilitate such monitoring and periodic recalibration [41], underscoring the practical utility of these models for clinical dose management despite their identified limitations.

### 4.2. Limitations of K-Factor Methods and Future Considerations

The limitations of K-factor methods were evident in our study. While we observed strong linear relationships between ED^ref^ and patient weight/BMI (R^2^ ≥ 0.84), K-factors, being population-averaged, inherently fail to fully capture these individual morphological variations. The systematic underestimation for women is a critical example of this limitation, suggesting that a single factor cannot adequately represent the varying radiosensitivity and anatomical differences between sexes [8]. This is further exacerbated in edge cases like obese patients, where increased scatter and altered photon attenuation are not accounted for by a simple conversion [49].

Furthermore, the reliance of K-factors on standardized phantoms means they may not capture unique individual organ geometries. Future refinements should therefore move beyond this simplified model. Promising avenues include developing sex-specific K-factors or, more powerfully, integrating patient-specific dose calculations based on CT raw data or machine learning models [40]. Such models could inherently correct for the complex interplay of patient morphology (sex, weight, BMI) and technical parameters (such as the strong tube current correlation found), thereby directly addressing the key sources of inaccuracy identified in these results.

## 5. Conclusions

The estimation of effective dose in CT has traditionally relied on complex Monte Carlo simulations, which are highly precise but unsuitable for routine clinical use. This study confirms that K-factor methods offer a simple, effective, and sufficiently reliable alternative for population studies and routine dose management.

These results quantify this reliability: the ED^rom^ method demonstrated clinically acceptable accuracy for the male cohort. For female patients, it introduced a modest median underestimation of −10%, which remains acceptable for population-level audits but indicates an area for future model refinement.

Despite this specific limitation, the overall performance level fully justifies the integration of this approach into clinical practice for essential tasks such as protocol optimization and compliance with Diagnostic Reference Levels (DRLs).

Finally, this work aligns with the principles of “green imaging”. The K-factor approach significantly reduces the demand for computational resources and energy consumption compared to traditional Monte Carlo methods, while facilitating large-scale dose audits.

Future studies should evaluate the robustness of these methods across a wider range of clinical scenarios (abdominal imaging, pediatrics) to refine their universal applicability.

## Figures and Tables

**Table 1:** The studied population, 1553 female patients and 2403 male patients, was partitioned by sex and into distinct BMI subgroups ranging from [15 17[ to [45 47[ kg/m^2^.

Population Investigated (n = 1553 Females + 2403 Males)									
BMI-group (kg/m^2^)	[15 17[	[17 19[	[19 21[	[21 23[	...	[39 41[	[41 43[	[43 45[	[45 47[
Number of Females	10	21	59	88	...	23	15	19	11
Number of Males	6	16	54	146	...	39	15	11	13

## Data Availability

The data can only be shared in Excel spreadsheet format upon request from the author, as they were extracted from the RDSRs located in the MSSSQ patient database.
